# Attitudes Toward School-Based Surveillance of Adolescents’ Social Media Activity: Convergent Parallel Mixed Methods Survey

**DOI:** 10.2196/46746

**Published:** 2024-02-06

**Authors:** Colin Burke, Cynthia Triplett, Caryn Kseniya Rubanovich, Melissa M Karnaze, Cinnamon S Bloss

**Affiliations:** 1 Herbert Wertheim School of Public Health and Longevity Science University of California San Diego La Jolla, CA United States; 2 Department of Sociology University of California San Diego La Jolla, CA United States; 3 Center for Empathy and Technology Institute of Empathy and Compassion University of California San Diego La Jolla, CA United States; 4 Joint Doctoral Program in Clinical Psychology San Diego State University San Diego, CA United States; 5 Joint Doctoral Program in Clinical Psychology University of California San Diego La Jolla, CA United States

**Keywords:** social media, surveillance, privacy, public health, students, schools, social media monitoring, SMM, school safety, mental health, adolescents

## Abstract

**Background:**

US schools increasingly implement commercially available technology for social media monitoring (SMM) of students, purportedly to address youth mental health and school safety. However, little is known about how SMM is perceived by stakeholders, including the students who are the focus of these efforts.

**Objective:**

We aimed to assess attitudes toward SMM in schools among 4 stakeholder groups and examine reasons for holding supportive, neutral, or unsupportive views toward the technology. We also sought to explore whether any differences in attitudes were associated with binary sex, race, ethnicity, sexual orientation, or gender identity.

**Methods:**

In October 2019, we conducted a convergent parallel mixed methods web-based survey of young adults (aged 18-22 y; n=206), parents (n=205), teachers (n=77), and school administrators (n=41) via Qualtrics web-based panels. We included Likert-type survey items to assess perceived benefits, risks, and overall support of SMM in schools and test for differences based on stakeholder group or demographic characteristics. We also included open-ended questions, and the responses to these items were analyzed using thematic content analysis of reasons given for holding supportive, neutral, or unsupportive views.

**Results:**

The tests of group differences showed that young adults perceived lower benefit (*P*<.001) as well as higher risk (*P*<.001) and expressed lower overall support (*P*<.001) of the use of SMM in schools than all other stakeholder groups. Individuals identifying as nonheterosexual also perceived lower benefit (*P*=.002) and higher risk (*P*=.02) and expressed lower overall support (*P*=.02) than their heterosexual counterparts; respondents who identified as people of racial and ethnic minorities also perceived higher risk (*P*=.04) than their White counterparts. Qualitative thematic content analysis revealed greater nuance in concerns about SMM. Specifically, the primary reasons given for not supporting SMM across all stakeholder groups were (1) skepticism about its utility, (2) perceived privacy violations, and (3) fears of inappropriate or discriminatory use of the data. Within the young adult group in particular, concerns were also raised about (4) unintended and adverse consequences, including the erosion of trust between students and school institutions and administrators, and the chronic adverse effects of constant or prolonged surveillance. Thematic analysis also showed that individuals in every stakeholder group who indicated overall support of SMM were likely to cite the potential for enhanced school safety as the reason. Young adults’ overall stances toward SMM were the most polarized, either strongly for or strongly against SMM, and responses from teachers indicated similar polarization but more often favored support of SMM in schools.

**Conclusions:**

This study found differing perspectives among stakeholder groups regarding SMM in schools. More work is needed to assess the ways in which this type of surveillance is being implemented and the range and complexity of possible effects, particularly on students.

## Introduction

### Background

In recent years, the United States has witnessed a troubling increase in both youth suicide [1] and incidents of school gun violence [2]. To address these concerning trends, public K-12 schools have implemented security measures, including the increased use of security cameras in schools as well as lockdown drills and protocols, based on data from the National Center for Education Statistics [1]. In addition, according to a recent survey, nearly 90% of school teachers reported that their school used technology during the 2021-2022 school year to track student activity on school-issued and personal devices, such as by accessing the content of students’ internet searches or remotely viewing students’ computer screens in real time [2]. As part of the increased surveillance of students, a growing number of schools have turned to commercially available social media monitoring (SMM) technology, which some companies claim will prevent harms on school campuses by monitoring students’ activity on the web, including, in some cases, activity that occurs outside of school hours [3-5].

SMM technology works by scanning public content posted by students on social media platforms such as Twitter, Facebook, and Instagram for certain words and phrases that might signal a threat of harm to oneself or others, in most cases without the explicit knowledge of students. The next step in the use of this SMM technology is the flagging of posts containing potentially problematic references to harmful behavior, such as suicide or self-harm, bullying, violence, or hate speech. When such posts are detected, the monitoring software alerts school officials, who can then notify teachers and parents or intervene by taking disciplinary measures or contacting school authorities or law enforcement. There is limited transparency regarding the specific workings of SMM (eg, algorithms, training data, and quality control) and limited evidence of its efficacy; for example, general overflagging of lesbian, gay, bisexual, transgender, queer, and similar minority (LGBTQ+)–related words has been noted across some SMM technologies, which raises issues of negative and disproportionate impact on certain groups of students and discrimination more broadly [3].

It has been difficult to determine exactly how many schools in the United States currently use SMM services. One of the first publicized uses of SMM technology was in September 2013, when the Glendale Unified School District in California hired the firm Geo Listening to monitor the social media content of 13,000 middle and high school students residing in their district [4,5]. Since 2013, the number of schools implementing SMM technologies has grown significantly. According to SmartProcure, a database for government purchase orders, in 2018, SMM services were purchased by schools to directly monitor the social media activity of >3 million students across 63 public school districts in the United States. This represented a 10-fold increase from 2013 when just 6 school districts were found by SmartProcure to have purchased such services [6]. More recently, Social Sentinel, a leading provider of SMM services, has claimed that it serves “thousands of schools in more than 35 states” [7]. The increasing frequency of anecdotal reports of SMM use in the media, usually in response to episodes of gun violence in schools, is consistent with this trend; for example, after the tragic shooting at Robb Elementary School in Uvalde, Texas, in May 2022, *The Dallas Morning News* reported that Uvalde was among at least 52 school districts in Texas alone that hired the firm Social Sentinel to monitor the social media content of its tens of thousands of students with the purported goal of preventing harm to students [7,8]. This statistic for just 1 SMM company may be a conservative estimate of school-based SMM across the United States.

Contrary to what might be expected, studies of general school surveillance practices suggest associations with decreased student perceptions of safety [9-12]; for instance, 1 study found that the use of security cameras outside of school was associated with higher perceived safety, but the use of cameras inside was associated with lower perceived safety, support, and equity [13]. Another study of American middle and high school students found that visible security measures (cameras, guards, and metal detectors) were associated with higher odds of students’ fear of exposures to violence, bullying and other harms at school [14]. Furthermore, a meta-analysis of qualitative studies found that students thought that the presence of closed-circuit television cameras often resulted in risky behaviors shifting from monitored areas to less-monitored areas (eg, hallways and restrooms) [15]. A 2021 survey conducted by the Center for Democracy and Technology also reported the “chilling” effects of web-based surveillance [16]. Specifically, 6 out of 10 students reported feeling uncomfortable expressing their true thoughts and feelings on the web if they knew they were being monitored. The report argued that chilling effects that curb exploration and self-expression could be especially problematic for minors and might also make students less likely to seek web-based resources for mental health, to their detriment [16].

Youth and minoritized communities are also likely to disproportionately experience unintended adverse effects of surveillance; for instance, the Center for Democracy and Technology report also speculated that web-based monitoring might pose a risk that LGBTQ+ students may be outed as a result of surveillance [16]. In addition, some minoritized youth have more fraught relationships with institutions such as law enforcement or disciplinary frameworks; for example, students of color are known to face higher rates and severity of punishment than their White peers, and thus, if surveillance leads to punishment, the effects of surveillance are more likely to fall on them. In fact, there is anecdotal evidence that school-based SMM has led to false positives [17], particularly with students of color. In addition, SMM algorithms may not accurately process content written in nonstandard English or languages other than English, and therefore SMM can disproportionately single out and label as dangerous students who are more likely to use nonstandard English or slang [6].

### Objectives

Although schools are increasingly deploying SMM technology, there has been no systematic assessment of how it is perceived by school stakeholders or how it might affect the students whom it purportedly aims to help. To address this gap, we conducted an exploratory survey assessing attitudes toward SMM in schools among 4 key stakeholder groups: school administrators, teachers, parents, and young adults. This survey included both closed-ended quantitative and open-ended qualitative questions to assess stakeholders’ attitudes. As a secondary aim, we also sought to statistically test for any differences in attitudes as a function of stakeholders’ self-reported gender, race, ethnicity, and sexual orientation.

## Methods

### Ethical Considerations

This study was reviewed and approved by the University of California, San Diego Office of Institutional Review Board Administration (191060) and received a waiver of signed consent. Each participant provided informed consent via radio button selection at the bottom of the web-based landing page that included written information about the study. Survey participants were compensated by Qualtrics.

### Recruitment

From October 7 to 15, 2019, we conducted an 8-minute web-based survey of young adults and parents via Qualtrics web-based consumer panels, as well as of teachers and administrators via Qualtrics web-based business-to-business panels. Participants on these panels are recruited from various sources, including website recruitment, member referrals, targeted email lists, gaming sites, customer loyalty web portals, permission-based networks, and social media. Qualtrics validates consumer panel members’ names, addresses, and dates of birth via third-party measures, and panel members are subject to additional quality control measures such as LinkedIn matching, telephone calls to the participant’s place of business, and other third-party verification methods (provided by companies such as TrueSample, RelevantID, and Verity). Although we originally desired to have an equal representation of teachers and administrators, this was not feasible owing to cost for the recruitment service. We also oversampled parents and young adults with the reasoning that, to date, these groups have been largely absent from dialogue and decision-making pertaining to SMM.

### Eligibility and Screening

Young adults were eligible if they were aged between 18 and 22 years and either a high school graduate or current high school student. Parents were eligible if they had children aged between 14 and 22 years. Teachers and administrators were eligible if they were employed in the education industry and were middle or high school teachers or administrators. For teachers and administrators, Qualtrics used a combination of the profiled information they had on file to target these professionals and screening questions at the beginning of the survey to confirm information for the specific survey respondents who qualified for the survey.

### Survey Design

The survey measure included (1) screening questions (4-7 items, dependent on skip logic), (2) basic demographic questions (8 items), and (3) questions soliciting views about SMM (9 items) that were modeled after another survey on public views of genome editing [18]. In the last category (Multimedia Appendix 1), there were 7 items that were measured on a 7-point Likert-type scale ranging from 1=strongly disagree to 7=strongly agree: 3 items asked about the perceived efficacy of SMM for addressing school-related violence, bullying, and mental health issues (ie, potential benefits of SMM); 3 items asked about the level of concern for SMM as it relates to privacy, data misuse, and discrimination (ie, potential risks of SMM); and 1 item asked about the overall level of support of the use of SMM in schools. There were also 2 open-ended questions: “Please describe how you feel about middle schools and high schools monitoring students’ social media activity.” “Is there anything else you would like to share about this issue?” This combination of closed- and open-ended questions reflects our use of a convergent parallel mixed methods design in which the quantitative and qualitative data collection occurred concurrently.

### Data Collection

We defined sample size quotas for the survey that aimed to collect responses from 200 young adults, 200 parents, 60 teachers, and 40 administrators. Qualtrics distributed the survey via a dashboard service whereby the survey would appear on a panel member’s dashboard if their profile indicated that they potentially met the inclusion criteria. We estimate that the survey was made available to between 14,400 to 16,000 individuals via this method, and 1600 individuals clicked a link to view the study information and consent page for the study. Of these 1600 individuals, 690 (43.13%) provided consent to participate in the study. Study data were collected and managed using the Qualtrics web-based survey platform. A *soft launch* to pilot-test the survey and check for any administration problems collected 30 responses that were used to establish quality benchmarks. Once data collection closed, responses were reviewed for completeness and quality in 2 phases. First, Qualtrics research panel staff filtered out all respondents who did not complete the survey or who completed the survey in less than half the median response time. Second, study team members filtered out any respondents who left gibberish in response to the open-ended questions.

### Data Analysis

#### Overview

We generated descriptive statistics to summarize and compare the sociodemographic characteristics of study participants. Quantitative data analyses were conducted using SPSS software (version 28.0; IBM Corp), and significance was set at *P*<.05 for all analyses. To enhance interpretability, an SMM benefits score was created by summing responses on the first 3 survey items pertaining to potential benefits from SMM (ie, to help address mental health, bullying, and threats of harm or violence; range: 3-21, with higher scores suggesting greater perceived benefits). Across this set of survey items, the Cronbach α value was .853, indicating high internal consistency. An SMM risks score was created by summing responses on the next 3 survey items pertaining to the potential risks of SMM (ie, it potentially violates privacy, leads to abuse or misuse of information, and leads to potential discrimination; range: 3-21, with higher scores suggesting greater perceived risks), and the Cronbach α value was .828. The final quantitative survey item assessed overall support of SMM in schools.

#### Quantitative Analyses

We used 1-way analysis of covariance and Bonferroni-adjusted post hoc pairwise comparisons to test for statistically significant differences among the 4 stakeholder groups on (1) SMM benefits, (2) SMM risks, and (3) overall support, controlling for sex (female vs male), race and ethnicity (non-Hispanic White vs all other groups), and sexual orientation (heterosexual vs all other groups). Partial eta–squared (η_p_^2^) was used as a measure of effect size, and the values of 0.01, 0.06, and 0.14 represent small, medium, and large effects, respectively [19]. To examine our second question regarding differences in the perceptions of SMM as a function of demographic characteristics, a series of independent samples 2-tailed *t* tests were conducted on the full sample to compare SMM benefits, SMM risks, and overall support of SMM by sex (female vs male), race and ethnicity (non-Hispanic White vs all other groups), and sexual orientation (heterosexual vs all other groups).

#### Qualitative Analyses

In analyzing the 2 open-ended questions, most participants answered such that their response to the second open-ended question was an extension of their answer to the first open-ended question. Thus, we considered responses to both items together and conducted thematic analysis with a contextualist lens [20]. Our inductive approach began by studying the short responses repeatedly to identify commonly discussed content, generating a list of 15 initial codes. A single coder then collated and refined the codes into a list of themes. As a final step, we integrated the data by merging the quantitative results with the qualitative results [21]. Specifically, the qualitative comments were sorted and read to identify similarities and differences within and among stakeholder groups, demographic groups, and levels of overall support of SMM in schools. In this way, we used triangulation to yield a more holistic understanding of the data and draw the conclusions set forth in the results presented.

## Results

### Sample Characteristics

A total of 690 individuals entered and consented to the survey. Of the 690 responses, 161 (23.3%) were identified as poor-quality completes and were removed from the data set. This yielded a final survey sample of 529 participants, which included young adults (n=206, 38.9%), parents (n=205, 38.8%), teachers (n=77, 14.6%), and school administrators (n=41, 7.8%). Table 1 provides descriptive statistics of the demographics of our sample.

**Table 1:** Descriptive statistics: Demographics of survey participants (n=529).

Variable		Administrators (n=41)	Teachers (n=77)	Parents (n=205)	Young adults (n=206)
Age (y), mean (SD)		46.9 (12.9)	41.1 (10.6)	48.8 (10.9)	21.0 (1.3)
Sex: female, n (%)		32 (78)	48 (62.3)	119 (58)	117 (56.8)
**Race and ethnicity, n (%)**					
	American Indian or Alaska Native	0 (0)	0 (0)	1 (0.5)	8 (3.9)
	Asian	1 (2.4)	3 (3.9)	10 (4.9)	12 (5.8)
	Black or African American	5 (12.2)	9 (11.7)	14 (6.8)	30 (14.6)
	Hispanic	4 (9.8)	4 (5.2)	20 (9.8)	49 (23.8)
	Native Hawaiian or other Pacific Islander	0 (0)	0 (0)	0 (0)	3 (1.5)
	Non-Hispanic White	30 (73.2)	62 (80.5)	170 (82.9)	132 (64.1)
	>1 race	3 (7.3)	2 (2.6)	6 (2.9)	11 (5.3)
	Other	2 (4.9)	1 (1.3)	4 (2)	10 (4.9)
Sexual orientation: heterosexual, n (%)		40 (97.6)	73 (94.8)	192 (93.7)	157 (76.2)

### Quantitative Results

#### Stakeholder Group Comparisons

**Figure 1:**
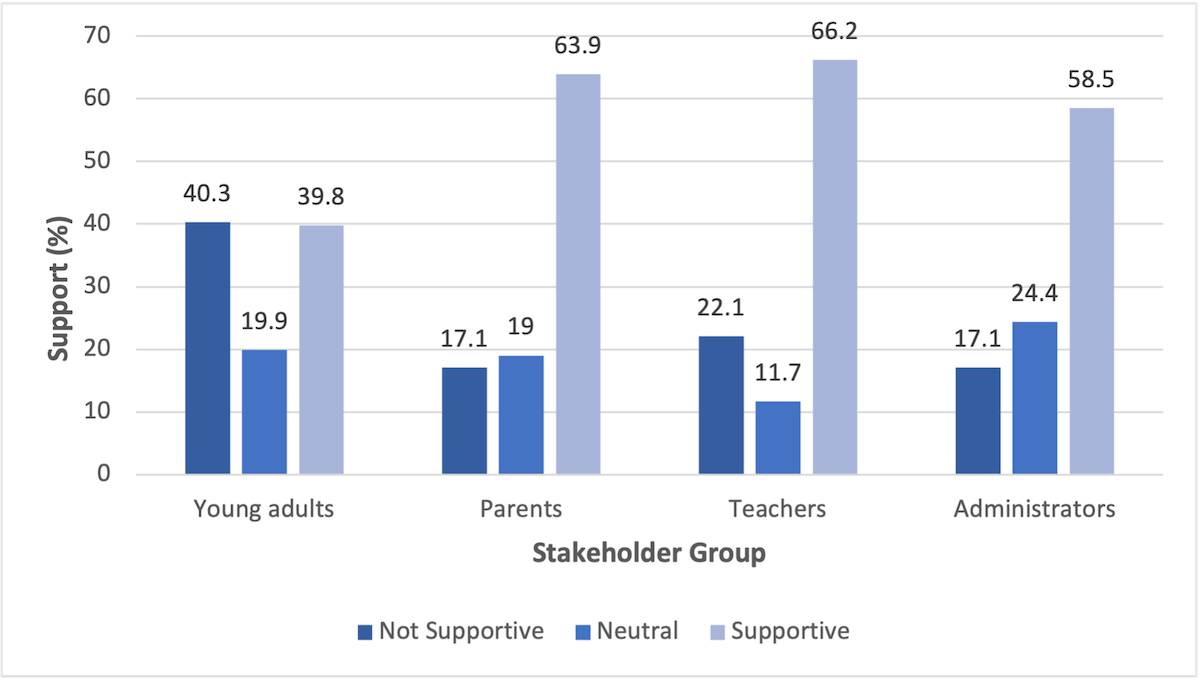
Overall opinion about the use of social media monitoring in schools by stakeholder group. Using data from question 7 of the survey, we collapsed all the agree categories (strongly agree, agree, and somewhat agree) into supportive and all the disagree categories (strongly disagree, disagree, and somewhat disagree) into not supportive; neutral refers to responses of neither agree nor disagree.

There were significant differences by stakeholder group in perceived SMM benefits, SMM risks, and overall support of SMM after controlling for sex, race, ethnicity, and sexual orientation. Table 2 provides the results of the analysis of covariance analyses, and Figure 1 shows the proportions within each group who were supportive, neutral, or unsupportive of SMM. Follow-up pairwise comparisons (data not shown) found that young adults were significantly different from all other groups and by comparison perceived lower benefit, higher risk, and less overall support of use of SMM in schools than parents, teachers, and administrators.

**Table 2:** Stakeholder group perceptions of social media monitoring (SMM) benefits, SMM risks, and overall support of SMM.

Variable	Administrators, mean (SD)	Teachers, mean (SD)	Parents, mean (SD)	Young adults, mean (SD)	*F* test (*df*)	*P* value	η_p_^2^
SMM benefits (range: 3-21)	16.93 (3.12)	16.92 (3.37)	16.39 (3.74)	14.05 (4.48)	12.68 (3,519)	<.001	0.068
SMM risks (range: 3-21)	12.20 (4.51)	12.83 (4.75)	13.60 (4.77)	15.21 (3.95)	7.11 (3,519)	<.001	0.039
Overall support of SMM (range: 1-7)	4.76 (1.58)	4.92 (1.71)	4.91 (1.64)	3.84 (1.81)	14.02 (3,519)	<.001	0.075

#### Demographic Group Comparisons

There were also significant differences by sexual orientation, race, and ethnicity. Specifically, nonheterosexual individuals perceived significantly lower benefit, higher risk, and less overall support of the use of SMM than their heterosexual counterparts. In addition, individuals identifying as people of racial and ethnic minorities perceived significantly lower benefit and higher risk than their non-Hispanic White counterparts. Tables 3, 4, and 5 provide the results of the 2-tailed *t* tests. There were no significant differences as a function of sex.

**Table 3:** Demographic group (sex) perceptions of social media monitoring (SMM) benefits, SMM risks, and overall support of SMM (n=526).

Variable	Sex, mean (SD)		*t* test (*df*)	*P* value
	Male (n=210)	Female (n=316)		
SMM benefits (range: 3-21)	15.21 (4.17)	15.87 (4.10)	−1.8 (524)	.08
SMM risks (range: 3-21)	14.10 (4.30)	13.93 (4.72)	0.41 (524)	.68
Overall support of SMM (range: 1-7)	4.38 (1.79)	4.56 (1.78)	−1.18 (524)	.24

**Table 4:** Demographic group (sexual orientation) perceptions of social media monitoring (SMM) benefits, SMM risks, and overall support of SMM (n=529).

Variable	Sexuality, mean (SD)			*t* test (*df*)		*P* value
	Heterosexual (n=462)	All other groups (n=67)				
SMM benefits (range: 3-21)	15.86 (3.95)	13.78 (5.14)	3.19 (77.67)		.002	
SMM risks (range: 3-21)	13.84 (4.55)	15.27 (4.53)	−2.4 (527)		.02	
Overall support of SMM (range: 1-7)	4.55 (1.76)	4.01 (1.90)	2.3 (527)		.02	

**Table 5:** Demographic group (race and ethnicity) perceptions of social media monitoring (SMM) benefits, SMM risks, and overall support of SMM (n=529).

Variable	Race and ethnicity, mean (SD)			*t* test (*df*)		*P* value
	Non-Hispanic White (n=349)	All other groups (n=180)				
SMM benefits (range: 3-21)	15.85 (3.96)	15.11 (4.53)	1.94 (527)		.05	
SMM risks (range: 3-21)	13.73 (4.68)	14.59 (4.28)	−2.08 (527)		.04	
Overall support of SMM (range: 1-7)	4.53 (1.82)	4.40 (1.72)	0.78 (527)		.44	

### Qualitative Results

#### Overall Stances Toward SMM and Stakeholder Group Differences

Generally, responses from the young adult cohort were the most polarized, with most respondents being either strongly in favor or strongly against the use of SMM in schools. Responses from teachers indicated similar polarization, but they were more commonly in favor of school-based SMM.

#### Reasons to Support SMM

Across all stakeholder groups, among those who indicated that they supported the use of SMM, the primary reason offered for this support was its potential utility to assist in identifying or preventing violence and bullying. One respondent noted as follows:

Kids are bullied every single day and are taking their lives. They won’t hardly talk to anyone and it mostly happens on Facebook or Instagram and things of that sort. I believe it would be a good idea to slightly monitor social media.

Young adult respondent 3MtgW

Another respondent stated that SMM might be a valuable service “if watching children’s social post[s] could save a child from bullying, suicide or abuse” (Parent respondent 23gc8). Surveillance, according to respondents, could be 1 tool in a school or school district’s safety toolbox as “an extra layer of protection to make sure the school is safe” (Teacher respondent YQzyo) and “no different [than] installing metal detectors to screen for weapons” (Administrator respondent 3MGxE). Justification for respondents’ support ranged from feeling that SMM was “a necessary evil” (Parent respondent 27JO1) to feeling that SMM was “absolutely necessary” (Teacher respondent 3fjYb) and “a good and wise thing to do” (Young adult respondent BKwWJ).

#### Reasons for Concern About SMM

##### Overview

Across the stakeholder groups, individuals who were unsupportive of SMM cited similar reasons for their stance. Specifically, our qualitative analysis showed that the primary reasons given for not supporting SMM that were cited across all stakeholder groups were (1) skepticism about its utility, (2) perceived privacy violations, and (3) fears of inappropriate or discriminatory use of the data captured in SMM reports. Importantly, the critics of SMM also felt that SMM in schools could lead to (4) unintended and adverse consequences, such as the erosion of trusted relationships among students, parents, and schools or the chronic adverse effects of constant or prolonged surveillance. We expand on each of these areas in the following subsections, and additional relevant quotes are provided in Textbox 1.

**Examples of reasons provided for not supporting social media monitoring (SMM).**

**Skepticism about utility of SMM**

- Administrators: “Sometimes the content posted can also be figurative speech so you can’t really determine if they mean it or not. Example: ‘I’m so going to kill you.’ 99% of the time that term is used jokingly.” (Administrator respondent 6JO0U)
- Teachers: “Well, I’m not sure how it would be done. It’s not easy to monitor...Hiring a social media monitoring staff seems just a bit too far for me. My school uses Twitter and Facebook to interact with students but there are so many app based forums which are far more popular with the youth. As is typical, us adults are ten years behind the kids in terms of tech.” (Teacher respondent 2YJac)
- Parents: “[I] post things on social media that I can understand but it may not exactly tell you who I am as a person. I’m not sure how true to light you can actually see a person as far as social media goes.” (Parent respondent 3GvrZ)
- Young adults: “[S]tudents would feel very violated and would try not to post revealing content or get around this by making another account with fake information.” (Young adult respondent 2rMEm)

**Perceived privacy violations**

- Administrators: “Invasion of privacy and over reach of responsibility.” (Administrator respondent 2CVC0)
- Teachers: “The harms and benefits of surveillance aren’t mutually exclusive. It’s a question of which are more important. In my opinion privacy and misuse are a bigger concern than mental health and bullying. There are other ways to deal with those problems. On the other hand, there are fewer ways to protect the students privacy and prevent schools from abusing their power. Who is watching the watcher?” (Teacher respondent 25G66)
- Parents: “What happens to the data and how are concerns then addressed! Schools often seem to do a poor job of intervening when kids are being bullied even without access to online social media accounts!” (Parent respondent 2vktc)
- Young adults: “I feel like it can help see bullying in schools and some threats to the school, but it does violate privacy. When a student steps into school their freedoms are revoked, but once they are out of school they have a right to privacy and monitoring would take away from that right.” (Young adult respondent 22Gtq)

**Fears of inappropriate or discriminatory use of data**

- Administrators: “I have concerns that middle and high school staffs are not fully equipped to deal with bullying and school violence. I fear that certain students from certain backgrounds will be discriminated against based on what they write/say on social media platforms.” (Administrator respondent 3241l)
- Teachers: “I am concerned how the information would be used—who would be ‘singled’ out for further actions?” (Teacher respondent 1GPQl)
- Parents: “I’m afraid schools will abuse it and students will get in trouble for every little thing school administration disapproves of.” (Parent respondent 3CUT0)
- Young adults: “I think it’d be a good idea but I also think that a lot of teachers would mistreat some students. I’ve had it happen to me before over something I didn’t even post and I can tell you it made me more depressed than before. The idea is good but you can’t trust teachers to be fair, they are only human and it could really hurt a student’s mental health when the teachers act like children themselves.” (Young adult respondent 2dM7k)

##### Skepticism About the Utility of SMM

The main question raised by those who did not support the use of SMM in schools was whether any SMM company, presumably operated by adults, could accurately and reliably catch troubling posts. Some felt that once students were aware of the monitoring, they would simply make their posts private, rendering the monitoring efforts ineffective. Stakeholders were also doubtful that teachers and administrators could correctly interpret students’ social media posts, given the loss of context and the challenge of deciphering irony in web-based messages. Finally, stakeholders questioned the overall feasibility of SMM because “adults are ten years behind the kids in terms of tech” (Teacher respondent 2YJac). On this point, another respondent wrote that “the threats are made through apps 9 times out of 10 where the message disappears” (Parent respondent yNHHU), referring to apps that allow users to send text messages that are automatically deleted after a period of time or once read by the recipient.

##### Perceived Privacy Violations

A major concern expressed by those who were unsupportive of the use of SMM was that monitoring would violate student privacy because “kids have the right to a life outside of school” (Teacher respondent 2fkRi) and such monitoring “removes a space where students can feel totally free to be themselves” (Young adult respondent 1mjXy). Other comments expressing concern ranged from those who simply stated that SMM was an invasion of privacy to those who felt discomfort with schools taking such actions:

[I]t feels a bit weird. Like an over reaching of boundaries. It just feels not right for schools to be monitoring personal social media accounts usually meant for friends or family.

Young adult respondent 3QF2F

The words “private” or “privacy” were explicitly mentioned in roughly a third of all young adult and administrator comments (72/206, 35% and 16/41, 39%, respectively), whereas parents and teachers mentioned these words less often (27/205, 13.2% and 15/77, 19%, respectively).

##### Fears of Inappropriate or Discriminatory Use of Data

The most frequently cited concern among those who were not supportive of SMM was the potential for SMM reports to be used in discriminatory ways (eg, discriminatory punishment). However, these concerns were raised primarily by young adults and parents, with teachers and administrators citing this concern in only 2 instances. Respondents worried that SMM might “become too far reaching and subjective” (Parent respondent 3iqKX). Young adults, in particular, feared that they would get in trouble for simply “posting what they feel like or how they feel” (Young adult respondent 1dnr8) “because staff at the school may not agree with posts or get offended by them and cause them to feel negatively toward that student and treat them unfairly” (Young adult respondent WjMZk). More broadly, respondents across all stakeholder groups recognized the possibility for SMM to exacerbate unconscious or conscious biases.

##### Unintended and Adverse Consequences

Finally, respondents wondered how the use of SMM might have some unforeseen and adverse impact:

It is a slippery slope...It seems like a good idea as far as safety, but I worry about what the information could be used for and if it will cause more trouble than good.

Administrator respondent 2SIjs

Young adult stakeholders, in particular, raised specific concerns about the potential for SMM to have the opposite of its intended effect (bold emphasis added by the authors); for instance, a young adult respondent noted the potential psychological and behavioral impact of SMM:

[I]*f I were to be monitored, I would simply not use social media at all. The idea of people overlooking my online presence is anxiety-inducing and should not be allowed.*

Young adult respondent 3lXio

Similarly, another young adult respondent stated as follows:

Monitoring students constantly can lead to a sense of paranoia as students are constantly being watched in real life, by their parents, teachers and if monitoring is enabled on social media.

Young adult respondent 1ilirF

A different young adult respondent brought up the potential chilling effect of SMM on students:

I feel like schools monitoring students’ social media activity is like being a helicopter parent which isn’t necessarily bad, but it may restrict the student’s freedom knowing they’re always watched, that if they say something someone doesn’t agree with, they may be punished.

Young adult respondent 2uy40

Another young adult respondent pointed out the potential strain on students’ relationship with educational institutions:

That’s a big overstep, also considering developmental psychology of that age group that seems like it would not go over well at all with the students and would brew animosity towards the schools.

Young adult respondent 32JeH

All these examples indicate that young adults felt that SMM in schools, contrary to its stated purpose, might increase feelings of anxiety and paranoia, potentially leading to detrimental mental health outcomes and ultimately worsening student relationships with teachers, administrators, and the school system overall.

## Discussion

### Principal Findings

This study assessed attitudes toward SMM in schools and identified similarities and differences across groups of young adults, parents, teachers, and school administrators, as well as across demographic groups. We found that the young adults we surveyed perceived lower benefit as well as higher risk and expressed lower overall support of the use of SMM in schools than all other stakeholder groups. In addition, individuals identifying as nonheterosexual also perceived lower benefit as well as higher risk and expressed lower overall support than their heterosexual counterparts. Respondents identifying as people of racial and ethnic minorities also perceived higher risk than those identifying as White. Qualitative thematic analysis highlighted the nuances of stakeholder attitudes and found that individuals in every stakeholder group who indicated support of SMM were likely to cite enhanced school safety as the reason. Individuals who were unsupportive cited skepticism about the utility of SMM, perceived privacy violations, and fears of inappropriate or discriminatory use of data. Young adults, in particular, also raised concerns about unintended consequences, including the erosion of trust between students and school institutions and the chronic adverse effects of constant or prolonged surveillance. Taken together, this study provides some of the first empirical documentation of stakeholders’ attitudes toward the use of SMM technologies in schools and is a first step toward generating needed discourse around this emerging technology.

Although we anticipated a priori that young adults would express the most unfavorable views of SMM, the qualitative responses we received indicated that this group thoughtfully considered potential benefits as well as potential drawbacks of SMM in schools. Specifically, young adults across the board, including those who were neutral or generally supportive of SMM, raised concerns about privacy and discrimination. This suggests that even young adults who favor school-based SMM may be concerned about potential harms to students, including that it could lead to negative mental health outcomes, the opposite of its intent. These findings suggest that young people do see problems or concerning trends in their schools and see a need for intervention but are skeptical about whether SMM is an appropriate or effective solution to such problems. The young people in our survey also demonstrated a keen awareness of trends in social media and web-based communication that SMM service providers and clients need to be aware of when using SMM and interpreting social media content.

The diverging viewpoints between young adults and the other stakeholder groups is also an essential finding because students are the primary targets of SMM; yet, they have had very little decision-making power in the implementation of these surveillance systems. The skepticism among young people toward these technologies further problematizes their general absence from the decision-making process, especially when schools that use SMM services often do so without students’ consent and, in some cases, without their knowledge [22]. Our results highlight how schools that consider or implement SMM should at a minimum engage in dialogue with students and recent graduates and consider how to make surveillance practices and policies more transparent. More research is also needed to better understand young adults’ concerns about currently evolving technologies and surveillance methods to minimize potential harm to students. This is particularly relevant for those who are not yet adults and may have fewer legal protections should school-based actions be taken against them based on social media data received from SMM companies. Similarly, constructing policies and ethical standards for SMM in schools would require bridging any gaps between the perceptions and knowledge of young adults and those of other stakeholder groups, perhaps by developing shared conceptual frameworks.

Expressed skepticism about SMM efficacy is also particularly salient, given that SMM continues to operate as a *quickly moving target*. The tragic school shooting in Uvalde, Texas, provides an unfortunate example of a case when SMM in schools did not function as claimed. *The Dallas Morning News* reported that according to records from GovSpend, an organization that tracks state and local government spending, the Uvalde Consolidated Independent School District was among at least 52 Texas school districts that hired Social Sentinel to monitor the social media activity of its tens of thousands of students [7,8]. However, like other SMM service providers, Social Sentinel only monitors public social media activity and, consequently, was unable to detect the shooter’s private communications related to the shooting [23]. The failure of SMM to prevent this tragedy has raised questions about the efficacy of such technologies and whether the potential harms of SMM might outweigh the potential good [24]. Our findings suggest that any cost-benefit analysis of SMM in schools must directly probe perceived costs and benefits from the members of all stakeholder groups and seek to recruit individuals across the spectrum of attitudes because, although there were commonalities in attitudes expressed across participants, the groups did diverge on issues. In particular, discrimination was more important to parents and young adults than to administrators, and the group approximating the population considered vulnerable of school-attending youth—young adults—provided richer descriptions of unintended consequences, of which other stakeholder groups and SMM companies need to be aware. It will also be important to gather insights from the public and individuals situated within the technology industry and predictive sciences who can provide expert opinion on what constitutes *efficacy* regarding purported SMM benefits.

We found that SMM perceptions also significantly differed by respondent sexual orientation such that nonheterosexual respondents saw fewer benefits (*P*=.002) and greater risks of SMM (*P*=.02), leading to less overall support of SMM (*P*=.02). LGBTQ+ individuals, as a group, have been reported to be frequent social media users [25], more so than heterosexual individuals [26] or the general public [27]. Previous literature has underscored the importance of social media for LGBTQ+ individuals. Specifically, social media is used as a space for identity exploration, social support, making platonic or romantic connections, and finding resources [28,29]. Moreover, social media has been described as a “safe space” [30] for LGBTQ+ youth. Given the levels of anonymity [31] or privacy settings [32] that social media can afford, LGBTQ+ individuals can manage how or whether to disclose their identities as well as express themselves more fully with less fear of stigma or marginalization than with in-person interactions. Through this lens, SMM might continue to disproportionately affect LGBTQ+ individuals and jeopardize the safety and anonymity they feel in using social media. We also found that the perceptions of SMM risks differed by race and ethnicity such that people racial and ethnic minorities respondents perceived greater risks. This finding may be explained by the “racial discipline gap” [33] or the disproportionate rate of school disciplinary sanctions against students of color. Given the long history of differential disciplinary treatment (eg, suspensions and expulsions) of students belonging to racial and ethnic minority groups compared with their non-Hispanic White counterparts, respondents may have concerns about the potential inequitable disciplinary actions taken as a result of SMM surveillance.

More broadly, some of these concerns have also been underscored by a recent US Congressional investigation [34] of 4 educational technology companies, which found that their surveillance platforms may be misused for disciplinary purposes, that surveillance often occurs around the clock (with alerts sometimes bypassing school personnel and going straight to law enforcement), and that parents are not adequately informed. This investigation concluded that “these surveillance products may continue to put students’ civil rights, safety, and privacy at risk” [34] and called on “the federal government...to track the potential impacts of student surveillance technology on students in protected classes...and work to ensure that products used by schools maintain student safety and privacy.” Our study seeks to answer this call to action by generating new insights about stakeholder perceptions of SMM. Moreover, although this study takes 1 step in this direction, more discussion among key stakeholder groups is essential to enhancing awareness and understanding of these technologies and their potential consequences. Failure to do so could have significant consequences, including the erosion of trust among stakeholders, such as students, parents, and educational institutions. Youth mental health and school safety are both urgent and increasingly complex societal challenges, and SMM represents an effort to look to science and technology for a solution. However, per the “technologies of humility” espoused by Jasanoff [35], we must reflect carefully on the ethical dimensions of this landscape and seek to understand and alleviate vulnerability to harm and be mindful of the distribution of risks and benefits.

The need for greater discourse in this area has also been amplified by the COVID-19 pandemic because the blurring of educational and digital spaces has led, and likely will continue to lead, to greater adoption of digital monitoring technologies. The expansion of school administrators’ guardianship and jurisdiction over students beyond school grounds and into digital spaces is likely to continue in this environment, meaning that critical scholarship and further investigation into these technologies are urgently needed. This urgency is further underscored by the lack of tangible and effective solutions to ongoing issues around youth violence, bullying, and suicidality. The immense pressure on schools to address such issues will likely lead to the further adoption of SMM without fully considering the potential consequences and harm that may result from such decisions.

This work has some limitations. First, we designed this as an exploratory survey, and thus the items were not validated for specific populations. Future surveys that use validated measures could more meaningfully probe associations between attitudes toward SMM and the characteristics of students. One future direction could be to use the insights from the qualitative data to inform the creation of more specific survey items assessing the perceived benefits and risks of SMM. Second, our findings reflect a sample of convenience, and future studies should seek to obtain nationally representative samples and samples with higher response rates. Third, although the data collected by our open-ended questions were valuable for our analysis, future studies might use in-depth interviews and focus groups to gain a deeper understanding of stakeholder attitudes and beliefs. Finally, this work did not survey current middle or high school students, which would be a fruitful approach in future work to gain more direct insight into young people’s perspectives and attitudes toward SMM.

### Conclusions

The results of this study reveal commonalities as well as divergences across stakeholder groups, both in surveyed attitudes regarding SMM in schools and open-ended responses provided directly by participants. The results also highlight the need for greater inclusion of individuals identifying as members of marginalized groups. Future research could examine what steps can be taken to foster greater inclusion of these groups in dialogue and decisions regarding the use of SMM in schools and investigate the real and potential harms and consequences of the use of SMM technologies for those being surveilled.

## Appendix

Multimedia Appendix 1 Social media monitoring exploratory survey.

## Abbreviations

LGBTQ+: lesbian, gay, bisexual, transgender, queer, and similar minority
SMM: social media monitoring
