# Supplementary material for: Attitudes Toward School-Based Surveillance of Adolescents’ Social Media Activity: Convergent Parallel Mixed Methods Survey
Source: JMIR Form Res. 2024 Feb 6;8:e46746. doi: 10.2196/46746 (PMC10879966; doi:10.2196/46746)
Supplement: Multimedia Appendix 1 [file formative_v8i1e46746_app1.docx]

**APPENDIX A**

***Social media monitoring (SMM) Exploratory Survey***

Schools have recently made attempts to address problems faced by their students, such as mental health issues, bullying, and violence. Because social media websites have become a common way for students to express themselves, social media has been viewed as a potential site for intervention and prevention of these issues. As a result, some schools have begun to use services from private companies that monitor students’ activity on social media. This has led to debate about whether schools, particularly middle schools and high schools, should use these services to monitor students’ activity on social media websites.

To take part in the debate, please indicate the degree to which you agree with the following statements ranging from 'Strongly disagree' to 'Strongly agree,' followed by any other comments that you might have about this issue.

1. **Monitoring students’ activity on social media websites will help middle schools and high schools recognize students’ mental health issues.**

🡨1------------------2-----------------3---------------------4---------------------5-----------------6----------------7🡪

Strongly Disagree Somewhat Neither Agree Somewhat Agree Strongly

Disagree Disagree nor Disagree Agree Agree

1. **Monitoring students’ activity on social media websites will help middle schools and high schools recognize bullying among students.**

🡨1------------------2-----------------3---------------------4---------------------5-----------------6----------------7🡪

Strongly Disagree Somewhat Neither Agree Somewhat Agree Strongly

Disagree Disagree nor Disagree Agree Agree

1. **Monitoring students’ activity on social media websites will help middle schools and high schools recognize threats of harm or violence to the school community.**

🡨1------------------2-----------------3---------------------4---------------------5-----------------6----------------7🡪

Strongly Disagree Somewhat Neither Agree Somewhat Agree Strongly

Disagree Disagree nor Disagree Agree Agree

1. **Monitoring middle school and high school students’ activity on social media websites violates students’ rights to privacy.**

🡨1------------------2-----------------3---------------------4---------------------5-----------------6----------------7🡪

Strongly Disagree Somewhat Neither Agree Somewhat Agree Strongly

Disagree Disagree nor Disagree Agree Agree

1. **Monitoring middle school and high school students’ activity on social media websites may lead schools to abuse or misuse the information that students post**.

🡨1------------------2-----------------3---------------------4---------------------5-----------------6----------------7🡪

Strongly Disagree Somewhat Neither Agree Somewhat Agree Strongly

Disagree Disagree nor Disagree Agree Agree

1. **Monitoring middle school and high school students’ activity on social media websites may lead schools to discriminate against certain students or groups of students.**

🡨1------------------2-----------------3---------------------4---------------------5-----------------6----------------7🡪

Strongly Disagree Somewhat Neither Agree Somewhat Agree Strongly

Disagree Disagree nor Disagree Agree Agree

1. **Middle schools and high schools should monitor students’ activity on social media websites.**

🡨1------------------2-----------------3---------------------4---------------------5-----------------6----------------7🡪

Strongly Disagree Somewhat Neither Agree Somewhat Agree Strongly

Disagree Disagree nor Disagree Agree Agree

1. **Please describe how you feel about middle schools and high schools monitoring students’ social media activity.**

Open Ended

1. **Is there anything else you would like to share about this issue?**

Open Ended
